# DDX4-*EGFP* transgenic rat model for the study of germline development and spermatogenesis ^†^

**DOI:** 10.1095/biolreprod.116.142828

**Published:** 2017-02-20

**Authors:** Kathrin Gassei, Yi Sheng, Adetunji Fayomi, Payal Mital, Meena Sukhwani, Chih-Cheng Lin, Karen A. Peters, Andrew Althouse, Hanna Valli, Kyle E. Orwig

**Affiliations:** Department of Obstetrics, Gynecology, and Reproductive Sciences, University of Pittsburgh School of Medicine, Magee-Womens Research Institute, Pittsburgh, Pennsylvania, USA

**Keywords:** VASA, DDX4, transgenesis, rats, germline, spermatogonial stem cells, spermatogenesis

## Abstract

Spermatogonial stem cells (SSC) are essential for spermatogenesis and male fertility. In addition, these adult tissue stem cells can be used as vehicles for germline modification in animal models and may have application for treating male infertility. To facilitate the investigation of SSCs and germ lineage development in rats, we generated a DEAD-box helicase 4 (DDX4) (VASA) promoter-enhanced green fluorescent protein (EGFP) reporter transgenic rat. Quantitative real-time polymerase chain reaction and immunofluorescence confirmed that EGFP was expressed in the germ cells of the ovaries and testes and was absent in somatic cells and tissues. Germ cell transplantation demonstrated that the EGFP-positive germ cell population from DDX4-EGFP rat testes contained SSCs capable of establishing spermatogenesis in experimentally infertile mouse recipient testes. EGFP-positive germ cells could be easily isolated by fluorescence-activated cells sorting, while simultaneously removing testicular somatic cells from DDX4-EGFP rat pup testes. The EGFP-positive fraction provided an optimal cell suspension to establish rat SSC cultures that maintained long-term expression of zinc finger and BTB domain containing 16 (ZBTB16) and spalt-like transcription factor 4 (SALL4), two markers of mouse SSCs that are conserved in rats. The novel DDX4-EGFP germ cell reporter rat described here combined with previously described GCS-EGFP rats, rat SSC culture and gene editing tools will improve the utility of the rat model for studying stem cells and germ lineage development.

## Summary Sentence

A DDX4 (VASA)-EGFP transgenic rat was produced to facilitate investigations of stem cells and germ lineage development.

## Introduction

The current understanding of spermatogonial stem cells (SSCs) and spermatogenic lineage development was derived from systematic histological studies of the testes in normal and chemically manipulated mice and rats [1–8]. Rats were the predominant animal model used by Clermont [5, 9] and Huckins [10–12] who are widely credited for the development of the A0/A1 and A_single_ (A_s_) models of spermatogenic lineage development, respectively. These models helped to fuel the ensuing 40 years of experimental inquiry [13–16]. Despite the important role the rat model played for the early studies on mammalian spermatogenesis, many investigators during the past 30 years have utilized mouse models to study stem cells and spermatogenesis because transgenic technologies and molecular reagents were more accessible for mice than for rats. Several germ cell-specific (GCS) reporter mouse lines have been published, including Acrosin [13], Protamine [14], synaptonemal complex protein 1 (*Sycp1*) [15], tissue-nonspecific alkaline phosphatase (*Tnap*) [16], POU domain, class 5, transcription factor 1 (*Pou5f1*) [17], phosphoglycerate kinase 2 (*Pgk2*) [18], neurogenin 3 (*Neurog3*) [19, 20], stimulated by retinoic acid gene 8 (*Stra8*) [21], general transcription factor IIA, 1-like (*Gtf2a1l*) [22], growth differentiation factor 9 (*Gdf9*), zona pellucida glycoprotein 3 (*Zp3*), and msh homeobox 2 (*Msx2*) [23], interferon induced transmembrane protein 3 (*Ifitm3*) [24], PR domain containing 1, with ZNF domain (*Prdm1*) [25], developmental pluripotency-associated 3 (*Dppa3*) [26], adhesion G protein-coupled receptor A3 (*Adgra3*) [27], *Ddx4* [28], *Nucleostemin* [29], nanos C2HC-type zinc finger 2 (*Nanos2*) and nanos C2HC-type zinc finger 3 (*Nanos3*) [30, 31], deleted in azoospermia-like (*Dazl*) [32], spermatogenesis and oogenesis specific basic helix-loop-helix 1 (*Sohlh1*) [33] and inhibitor of DNA binding 4 (*Id4*) [34]. In addition, mouse transgenic and knockout models have unequivocally demonstrated the functional importance of specific genes (e.g., *Zbtb16* [35, 36], glial cell line derived neurotrophic factor family receptor alpha 1 (*Gfra1*) [37], ets variant 5 (*Etv5*) [38], *Sohlh1* [33, 39], spermatogenesis and oogenesis specific basic helix-loop-helix 2 (*Sohlh2*) [40], forkhead box O1 (*Foxo1*) [41], *Sall4* [42], *Nanos2* [30], *Nanos3* [30], piwi-like RNA-mediated gene silencing 1 (*Piwil1*) [43], *Dicer* [44, 45], RB transcriptional corepressor 1 (*Rb1*) [46, 47], cAMP responsive element modulator (*Crem*) [48]) for SSCs and spermatogenesis. These models have provided valuable insights into sex-specific germ lineage development, spermatogonial self-renewal and differentiation, meiosis, and spermiogenesis. To gain insight into rat spermatogenesis, the data derived from transgenic mouse studies have then been extrapolated, but confirmation of the data is limited due to the limited availability of transgenic rat models. However, the recent advent of rat embryonic stem cells and novel gene editing tools (e.g., TALENs, ZFNs, and CRISPR/Cas9) [49–52] provide new opportunities to expand the use of the rat model in biomedical research and build on the strong foundation of historical physiological and morphological data that are available for this species.

There are several features that favor rats for fundamental and biomedical research. For example, surgical and physiological experimentation as well as repetitive sampling is easier in rats because of their relatively larger body size and blood volume. Litters are usually larger in rats than in mice, which facilitates animal husbandry and accelerates generation of experimental animals. Rats are approximately ten times larger than mice, therefore samples for genomic and proteomic downstream applications that require large amounts of nucleic acids or proteins are easier to obtain from rats. Similarly, the larger size facilitates the isolation of rare cell types from whole organs (e.g., adult tissue stem cells). Rat testes are ten times larger than mouse testes and contain more than 100 times more SSCs [53].

Here, we report the generation and characterization of a GCS transgenic reporter rat. This model will be useful for the study of germ lineage development and will facilitate tracking of donor-derived cells after transplantation. We used the Ddx4 (formerly known as *Vasa)* gene promoter [28] to drive enhanced green fluorescence protein (*Egfp*) reporter gene expression in germ cells of Sprague-Dawley (SD) rats. The *Ddx4* gene encodes a conserved member of the DEAD box helicase family and is specifically expressed in the germline of Drosophila [54], zebrafish [55], mice [56, 57], rats [58, 59], monkeys [60, 61], and humans [62]. In mice, DDX4 is expressed in primordial germ cells that have populated the gonadal ridges [63], whereas in rats DDX4 is upregulated earlier and was observed in migrating primordial germ cells (PGCs) [59]. Expression is sustained throughout germ cell development and DDX4 was observed in postmeiotic spermatids and oocytes in adult mice [57] and rats [64]. Targeted mutagenesis of the *Ddx4* locus resulted in mutant mice with sex-dependent reproductive defects. Male homozygous mutants are azoospermic due to a spermatogenic block in premeiotic spermatocytes that fail to progress through meiosis and undergo apoptosis [65]. Interestingly, female mutants were fertile and did not exhibit a germ cell phenotype. This suggests a sex-dependent role for DDX4 in rodents. Here, we characterize reporter gene expression in the developing germlines of DDX4-EGFP rats to provide a basis for understanding how this transgenic model might be deployed for investigation of germ lineage development, SSCs, and spermatogenesis.

## Material and methods

### Animals

All animal procedures were approved by the Institutional Animal Care and Use Committee of the University of Pittsburgh and Magee-Womens Research Institute in accordance with the National Institutes of Health Guidelines for the Care and Use of Laboratory Animals (Assurance # 3654-01). Animals were housed in the laboratory animal facility at Magee-Womens Research Institute.

### Generation of DDX4-EGFP transgenic rats

The *Ddx4* promoter was isolated from the Ddx4-Cre plasmid described by Gallardo and colleagues [28] using *PacI* and *SalI* restriction enzyme digestion. The promoter was inserted into the PacI/SalI sites of the mammalian expression vector pEGFP-ps (Clontech, Mountain View, CA). We then inserted an *Egfp-SV40pA* fragment into *XhoI/PacI* sites of the *pDdx4-Egfp* plasmid. The *Ddx4-Egfp-Sv40pA* (termed *Ddx4-Egfp* from this point forward) fragment was excised by *SalI* digestion and gel purified for injection into the male pronucleus of fertilized rat oocytes. Transgenic services were provided by the Genome Editing, Transgenic and Virus Core Facility of Magee-Womens Research Institute (http://www.mwrif.org/125).

Female SD rats (4–5 week old) were superovulated with intraperitoneal (i.p.) injection of pregnant mare serum gonadotropin (20 IU, i.p.; Calbiochem, Rockland, MA, No. 367222), followed 48 h later by i.p. injection of human chorionic gonadotropin (20 IU, i.p.; Sigma, St. Louis, MO, No.CG5). Females were housed with fertile SD males overnight and fertilized oocytes were collected the following morning. The linearized and purified *Ddx4-Egfp* plasmid (2 ng/μl) was injected into the male pronucleus of one-cell rat embryos. Embryos were cultured overnight in potassium simplex optimized medium (KSOM) (Millipore, Billerica, MA, No.MR-020P-D) supplemented with essential amino acids (Life Technologies, Grand Island, NY, No.11130-051). Embryos that developed to the two-cell stage were transferred into the oviducts of pseudopregnant recipient females. Pseudopregnancy was induced in embryo transfer recipient females by breeding with vasectomized SD males the night before transfer. Pups born after embryo transfer were screened for the presence of the *Ddx4-Egfp* transgene by polymerase chain reaction to detect Egfp cDNA (forward primer 5΄-ccacatgaagcagcacg-3΄ and reverse primer 5΄ gctttacttgtacagctcg-3΄).

### Quantitative real-time polymerase chain reaction

Total RNA from ovaries, testes, livers, kidneys, spleens, and lungs was isolated from ∼250 mg tissues using the Qiagen RNeasy Midi Kit (Qiagen, Valencia, CA, No.75144) according to the manufacturer's instructions. Total RNA from intestines, hearts, brains, and skeletal muscles was isolated from ∼100 mg tissues with Trizol (Life technologies, No.15596). Organs were dissected from three adult F4 generation offspring (>8 weeks of age). As controls, total RNA was isolated from three wildtype animals. The total RNA concentration was determined with a Thermo Scientific NanoDrop 2000c UV-Vis Spectrophotometer (Thermo Fisher, Pittsburgh, PA), and 5 μg total RNA from each tissue were treated with RNAse-free DNAse (2 units/μg RNA, Promega, Madison, WI, No. M6101) at 37°C for 60 min to eliminate any genomic DNA that might contaminate the sample. Next, 200 ng treated RNA was reverse transcribed with 200 units SuperScript III Reverse Transcriptase (Life Technologies, No. 18080-044) in 20 μl final volume at 50°C for 60 min in the presence of 40 units RNase Inhibitor (Life Technologies, No. 10777-019) using Oligo(dT)20 Primers (Life Technologies, No. 18418-020). For no reverse transcriptase (RT) controls, enzyme was replaced with double distilled H_2_O for each sample.

For SYBR Green quantitative polymerase chain reaction (PCR), 0.5 μl cDNA was used as template in a final volume of 10 μl per reaction. Polymerase chain reaction reactions were performed in triplicate for each tissue sample. EGFP primers were forward, 5΄-GAACGGCATCAAGGTGAACT-3΄, and reverse, 5΄-TGCTCAGGTAGTGGTTGTCG-3΄. As internal control, rat-specific ß-actin was amplified from each sample with primers forward, 5΄-CCCTGTGCTGCTCACCGAGG-3΄ and reverse, 5΄-GGCTACGTACATGGCTGGGGTG-3΄. Real-time PCR was performed on the 7900 HT Fast Real-Time PCR System (Applied Biosystems from Life Technologies) with SDS 2.4 software. Relative EGFP expression for each tissue was estimated using the delta-deltaCt (ΔΔCt) method.

### Statistics

The ΔCt (average GFP Ct – average β-actin Ct) was calculated for each tissue from all replicate experiments. EGFP expression in each tissue was then compared to testis (reference group) by calculating ΔΔCt (ΔCt[tissue] – ΔCt[testis]). Repeated-measures analysis of variance (ANOVA) was used to compare expression across different tissues. *P*-values less than 0.05 were considered statistically significant. Statistical analyses were performed using SAS version 9.4 (Cary, NC). Two-tailed Student's *t*-tests were performed to compare EGFP expression between transgenic testis and wildtype testis, and between transgenic ovary and wildtype ovary. *P*-values less than 0.05 were considered statistically significant.

### Fresh tissue fluorescent imaging of enhanced green fluorescence protein expression

Testes, ovaries, livers, spleens, kidneys, hearts, skeletal muscles, brains, lungs, and intestines were excised from a total of 19 transgenic rats at varying ages (1–6 months), rinsed with cold Dulbecco phosphate-buffered saline (DPBS, Life Technologies No. 14200-166), and examined with a stereomicroscope. Animals from several generations (F1, F2, F3, and F4) were included in this study. Tissues were slightly dried on tissue paper and imaged using a Nikon SMZ1500 Stereomicroscope equipped with a Fluorescein isothiocyanate (FITC)/Tetramethylrhodamine (TRITC) dual-emission filter, XCite Fluorescence Illumination System, and a SPOT digital camera. Bright-field and fluorescence images were captured from each tissue using the same exposure times for each tissue to compare endogenous EGFP signal in the various tissues. Tissues from wildtype litter mates were observed as controls. Tissues were processed within 15 min after dissection.

### Immunohistochemistry

Tissues were excised from a total of 21 4–6-week-old transgenic rats and fixed in 4% paraformaldehyde in DPBS overnight at 4°C. For developmental studies, male and female gonads were dissected from at least three pups at postnatal day 1, 8, and 14 and fixed as described above. All tissues were extensively washed with cold DPBS before processing for paraffin embedding. Tissue sections (5 μm) were collected on glass slides for immunohistochemistry. Sections were deparaffinized in a descending ethanol series, and antigen retrieval was performed in antigen retrieval buffer (10 mM Sodium Citrate, 0.05% Tween-20, pH 6) at 97.5°C for 30 min. Sections were incubated with a blocking buffer (5% goat serum, 3% bovine serum albumin, and 0.1% Triton X-100 in DPBS) for 30 min at room temperature to block unspecific binding sites. As primary antibodies, mouse monoclonal anti-EGFP (clone JL-8, Clontech, No.632381, diluted 1:500), polyclonal rabbit anti-DDX4 (Abcam, Cambridge, MA, No.ab13840, diluted 1:400), polyclonal goat anti-ZBTB16 (R&D Systems, No.AF2944, diluted 1:50), or monoclonal mouse anti-SALL4 (Abcam, Cambridge, MA, No.ab57577, diluted 1:200) were incubated with tissue sections over night at 4°C. After three washes with DPBS containing 0.1% Tween-20, secondary goat antirabbit AlexaFlour-568 and goat antimouse AlexaFluor-488 conjugated IgG (Life Technologies, 1:1000 diluted) were applied to sections and incubated at room temperature for 45 min. Sections were washed and mounted in Vectashield medium with 4΄,6-diamidino-2-phenylindole (DAPI) (Vectorlabs, Burlingame, CA, No.H-1200) for fluorescence imaging. Sections were imaged using a Nikon E600 fluorescence microscope with FITC/TRITC dual-emission filter, XCite Fluorescence light source, SPOT digital camera, and MetaVue software.

### Preparation of testicular single-cell suspension for germ cell transplantation, immunocytochemistry, and fluorescence-activated cells sorting

Testes from at least three juvenile DDX4-EGFP transgenic rats (6–15 days old) per experiment were dissected into ice-cold Hanks balanced salt solution (Life Technologies, No.24020-117) and the tunica albuginea was carefully removed. Tubules were dispersed using fine forceps, transferred to 1 mg/ml Collagenase IV (Sigma, No.C5138) solution, and digested at room temperature for 5 min with gentle agitation. Tubules were allowed to settle by gravity sedimentation and the supernatant containing interstitial cells was removed. Tubule fragments were then digested in 0.25% Trypsin-EDTA (Life Technologies, No.25200-114) containing 7 mg/ml DNase I (Sigma, No.DN25) for 5 min at 37°C. To achieve a single-cell suspension, tubule fragments were pipetted up and down until the solution reached a turbid appearance. Digestion was stopped with 10% volume fetal bovine serum (FBS, Fisher Scientific, No.SH30070 03), and the suspension was filtered through a 40-μm cell strainer to remove any remaining tubular fragments and clumps. Cell viability was determined by trypan blue exclusion, and cells were counted using a hemacytometer. Cell suspensions were analyzed by immunocytochemistry. Briefly, 10-μl droplets of the cell suspension were spotted on Superfrost microscope slides, fixed with excess ice-cold methanol or ethanol:acetic acid (7:1) mix and air dried on the slides. Immunocytochemistry is described below.

For germ cell transplantation, the final concentration was adjusted to 10 × 10^6^ cells/ml in minimum essential medium α (MEMα; Life technologies, No.12561-072) containing 10% FBS and 10% trypan blue. Of this solution, 7 μl was injected via the efferent ducts into the seminiferous tubules of each testis of four infertile busulfan-treated recipient nude mice using an Eppendorf NK2 Transferman micromanipulator and Eppendorf femtoject injector as described previously [61]. To allow for development of donor-derived spermatogenesis in recipient testes, nude mice were housed for 3 months. Recipient testes were recovered and tubules were isolated by removing the tunica albuginea and carefully dispersing the tubules with fine forceps. Tubules were examined for EGFP-positive colonies of spermatogenesis under a Nikon SMZ1500 Stereomicroscope equipped with a FITC/TRITC dual-emission filter, XCite Fluorescence Illumination System, and a SPOT digital camera.

Fluorescence-activated cells sorting (FACS) was performed on a FACSAriaII cell sorter (Beckton Dickinson) at the flow cytometry core facility at McGowan Institute for Regenerative Medicine, Pittsburgh. The cells were diluted in DPBS containing 5% FBS and kept on ice and covered from light. EGFP-positive cells were identified by comparison with single-cell suspensions obtained from wildtype littermate controls. Sorting gates were established based on EGFP expression and exclusion of dead cells stained with propidium iodide and exclusion of cells exhibiting autofluorescence.

### Cell culture

EGFP-positive cells isolated by FACS were cultured as described by Ryu and colleagues [66] with slight modifications. Briefly, mitomycin-treated (10 μg/ml) STO (SIM mouse embryo‐derived, thioguanine and ouabain resistant) feeder cells were plated at a density of 5 × 10^4^ cells/cm^2^ in 24-well culture plates that were precoated with 0.1% gelatin. After FACS, approximately 1–5 × 10^5^ cells were plated per well. For long-term culture, rat serum-free medium was prepared that contained MEMα (Life Technologies, No.12561) supplemented with 0.6% BSA (MP Biomedicals, No.810661), 50 units/ml Penicillin/50 μg/ml Streptomycin (Life Technologies, No.15140), 100 μg/ml transferrin (Sigma, No.T1283), 15.2 μeq/l free fatty acids [palmitic acid, palmitoleic acid, stearic acid, oleic acid, linoleic acid, linolenic acid at mM proportions 31:2.8:11.6:13.4:35.6:5.6 for 100 mleq/l ethanol stock solution], 6 × 10^−8^ M sodium selenite (Sigma, No.S5261), 2 mM L-Glutamine (Life Technologies, No.25030), 100 μM beta-mercaptoethanol (Sigma, No.M7522), 25 μg/ml insulin (Sigma, No.I1882), 10 mM Hepes (Life Technologies, No.15630-080), 120 μM putrescine (Sigma, No.P5780), 40 ng/ml human glial cell line-derived neurotrophic factor (GDNF; R&D Systems), 300 ng/ml rat GFRα1 (R&D Systems), 1 ng/ml basic fibroblast growth factor (bFGF; BD Biosciences), and 10% (vol/vol) water. Media was changed every 48 h, and cells were passaged at a ratio of 1:1.5–1:2 every 7 days.

For in-well immunocytochemistry, cells were rinsed with cold DPBS and fixed in 1% paraformaldehyde (PFA) at 37°C for 15 min and then washed three times with cold DPBS containing 0.1% TritonX-100 (DPBS-Tx). Immunocytochemistry was performed as described below.

### Immunocytochemistry

Cells fixed on Superfrost glass slides and were rehydrated with DPBS for 3 min. Cells on glass slides and in culture wells were then blocked with goat serum in DPBS for 30 min at room temperature. Primary antibodies used to analyze single-cell suspensions and cell cultures were polyclonal rabbit anti-SALL4 (Abcam, No.ab29112; 1:400), rabbit anti-DDX4 (Abcam, No.ab13840; 1:400), rabbit anti-ZBTB16 (Sigma, Atlas Antibodies, No.HPA001499; 1:50), and mouse anti-EGFP (JL-8, Clontech No.632381; 1:100). Primary antibodies were incubated for 60 min at room temperature. After washing three times for 3 min in DPBS plus 0.1% Tween-20, goat-antirabbit AlexaFluor568 and goat-antimouse AlexaFluor488 secondary antibodies were diluted 1:1000 and incubated with the cells for 45 min at room temperature. Slides were washed and mounted with Vectashield containing DAPI for microscopy.

## Results

### Generation of DDX4-EGFP transgenic rats

A total of 467 embryos were transferred to 12 pseudopregnant recipient rats. Overall, 7 litters (58.3%) with a total of 41 pups (8.8%) were born. We identified one female founder mouse (#2738, 2.4% transgenic efficiency) that carried the *Ddx4-Egfp* transgene (Supplementary Figure S1). We established a transgenic line by breeding this founder female with wildtype SD male rats. One male F1 offspring (#2832) was then subsequently used to propagate the transgenic line for future use (line DDX4-EGFP-2832).

### Gonad-specific reporter gene expression in DDX4-EGFP rats

**Figure 1.**
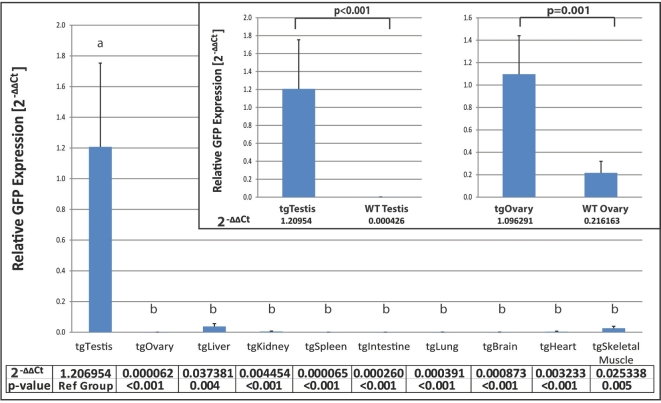
*Egfp* mRNA expression in testis and ovary compared to somatic tissues in DDX4-EGFP transgenic rats. Fold changes of *Egfp* mRNA levels (2^−ΔΔCt^) in selected organs are shown. The deltaCt (*Egfp* Ct – *Actb* (*β-actin*) Ct) was computed for each tissue on each animal. Repeated-measures ANOVA was used to determine whether the deltaCt values were significantly different across tissues. All tissues were compared to the testis (reference group) by calculating delta-delta Ct values (dCt(tissue) –dCt(testis)). *Egfp* expression in testis and ovary was compared between wildtype and transgenic animals using two-tailed Student's *t*-test. *P*-values less than 0.05 were considered statistically significant and are denoted by different letters.

Levels of reporter gene expression in a panel of tissues from F3 offspring of the established DDX4-EGFP transgenic line were determined by qualitative real-time PCR (Figure 1). All expression data were normalized to ß-actin mRNA levels as internal control. Analyzing ΔΔCt values, we observed that Egfp mRNA was present in the testis of transgenic males at levels significantly higher than in all other tissues tested, including wildtype testis (*P* < 0.001).

**Figure 2.**
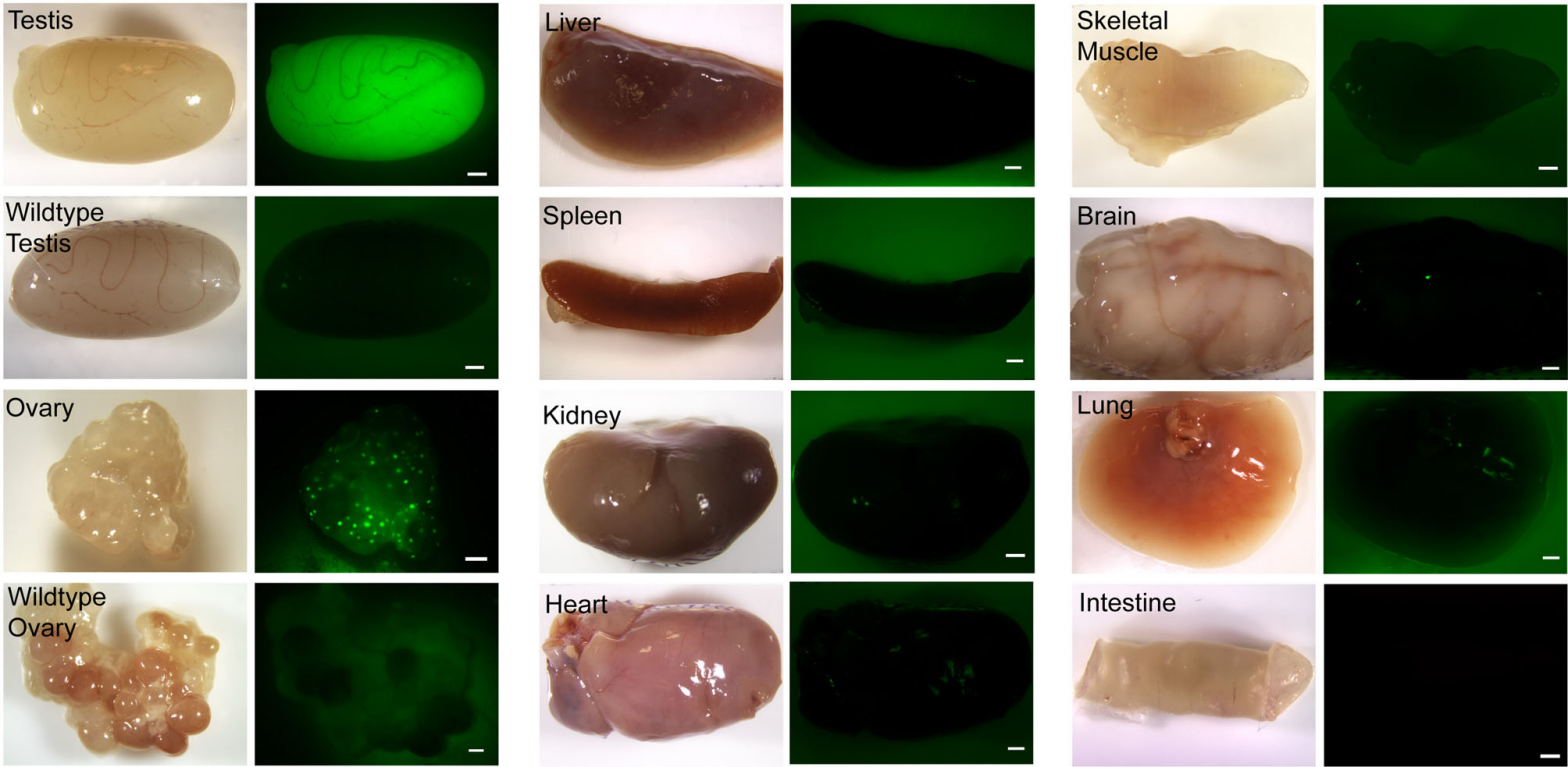
Live imaging evaluation of endogenous EGFP expression in selected organs of DDX4-EGFP transgenic rats. Representative bright-field (left) and fluorescent (right) images of selected organs from DDX4-EGFP transgenic rats are shown. Wildtype testes and ovaries served as negative controls. Strong endogenous EGFP expression was observed in transgenic testes. In transgenic ovaries, EGFP was observed in oocytes that are visible as small round green fluorescent dots. Somatic tissues did not express EGFP. Scale bar = 1 mm.

We used live imaging of fresh tissues to determine whether EGFP fluorescence could be detected using an epifluorescence dissecting microscope (Figure 2). Strong green fluorescence was consistently observed in the testes of DDX4-EGFP males (F1, F2, F3, F4 generation). In DDX4-EGFP transgenic ovaries, EGFP expression was observed in individual oocytes but not in the somatic cells of the follicles. Green fluorescence was not evident in ovaries and testes from wildtype littermate controls or in any of the somatic tissues.

**Figure 3.**
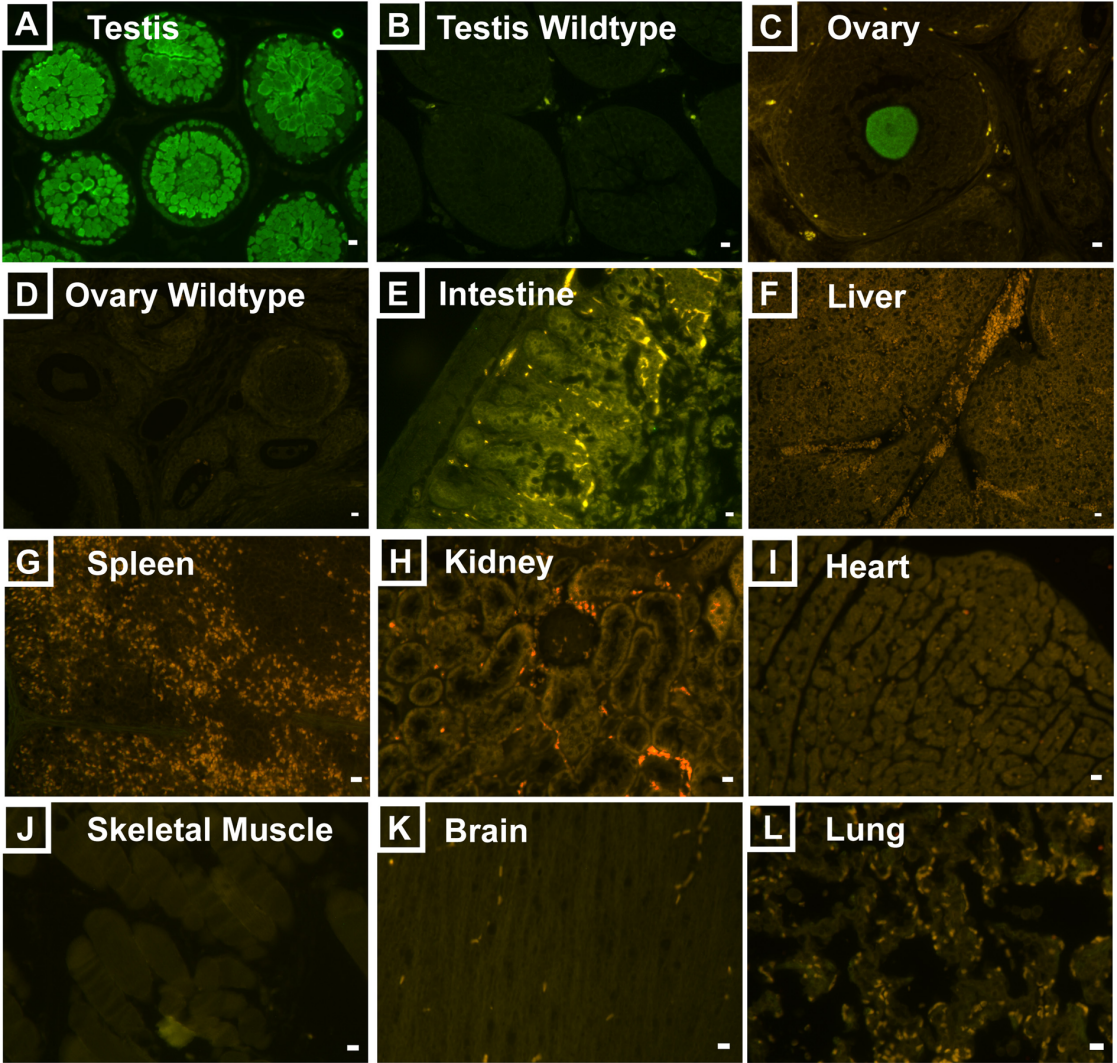
EGFP immunohistochemistry of various tissues from F3 offspring. Paraffin embedded tissue sections were stained with anti-EGFP antibody. Only germ cells in DDX4-EGFP testes (A) and ovaries (C) were EGFP positive. No staining was evident in wildtype testis (B) and ovary (D) sections, or any of the other organs that were evaluated (E–L). Sections were not counterstained with DAPI to allow observation of the different levels of autofluorescence in intestine, spleen, lung, and kidney tissue. Scale bars = 10 μm.

The epifluorescence results were confirmed by immunofluorescence staining of PFA-fixed, paraffin-embedded tissue sections from DDX4-EGFP rats using an anti-EGFP antibody (Figure 3). EGFP protein was confined to germ cells in the testes of DDX4-EGFP rats, but was not detected in the testes of wildtype from littermate controls (Figure 3A and B). Likewise, EGFP was observed in oocytes of the transgenic ovary but not in the wildtype ovary (Figure 3C and D). No EGFP was observed in any of the other organs examined (Figure 3E–L), but some autofluorescence was present in intestine, spleen, and kidney tissue. Autofluorescence appears red or yellow when using the FITC/TRITC dual emission filter, while EGFP fluorescence remains green.

### Enhanced green fluorescence protein protein expression in male and female germ cells

**Figure 4.**
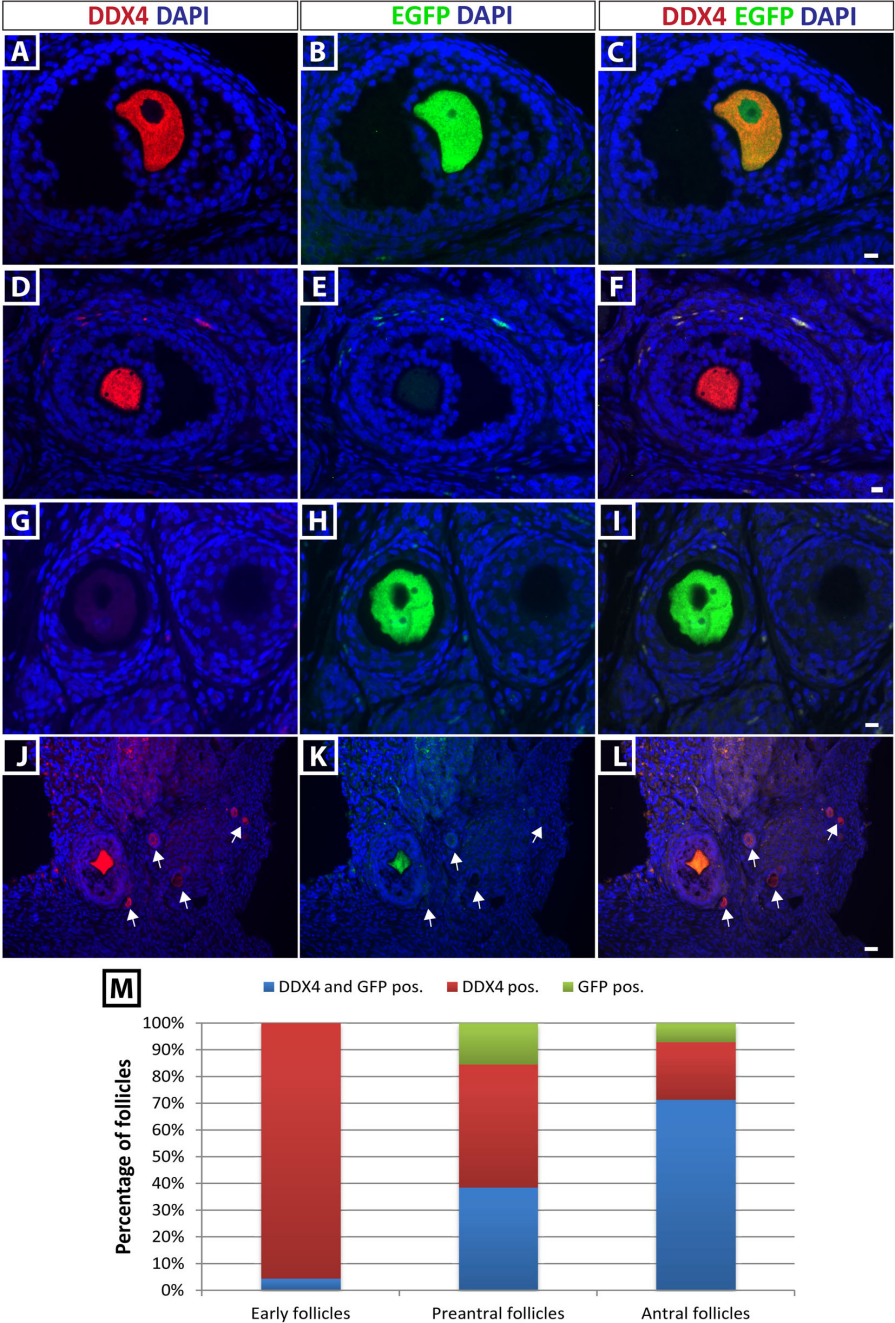
Mixed EGFP expression in oocytes of DDX4-EGFP transgenic rats. EGFP expression in ovaries was determined by coimmunofluorescence staining for DDX4 and EGFP in paraffin-embedded ovarian sections. (A–C) DDX4-positive, EGFP-positive rat oocyte. (D–F) Not all oocytes in DDX4-EGFP ovaries expressed EGFP. Panel E shows the lack of EGFP expression in an oocytes marked by DDX4 staining. (G–I) DDX4 expression is not consistent in all oocytes from 4-week-old rats as shown in panel G. EGFP was expressed in a DDX4-negative oocyte. Scale bars = 10 μm. (J–L) EGFP was not detectable in the majority of early (primordial/primary) DDX4-positive follicles (Arrows). Scale bar = 30 μm. Quantification of the DDX4 and EGFP costaining is represented in M.

To confirm the presence of EGFP protein in the predicted gonadal cell type (germ cells), we performed coimmunofluorescence staining for EGFP and DDX4 on PFA-fixed, paraffin-embedded gonadal tissue sections using rabbit anti-DDX4 IgG and mouse anti-EGFP IgG. In the ovary, a mixed reporter protein expression in oocytes was observed (Figure 4). While 38% of preantral follicles and 71% of antral follicles coexpressed DDX4 and EGFP protein (Figure 4A–I and M), only 4% of early follicles (primordial + primary) were positive for both (Figure 4J–M). Interestingly, we also observed oocytes that expressed the EGFP protein in the absence of DDX4 protein (Figure 4G–I).

**Figure 5.**
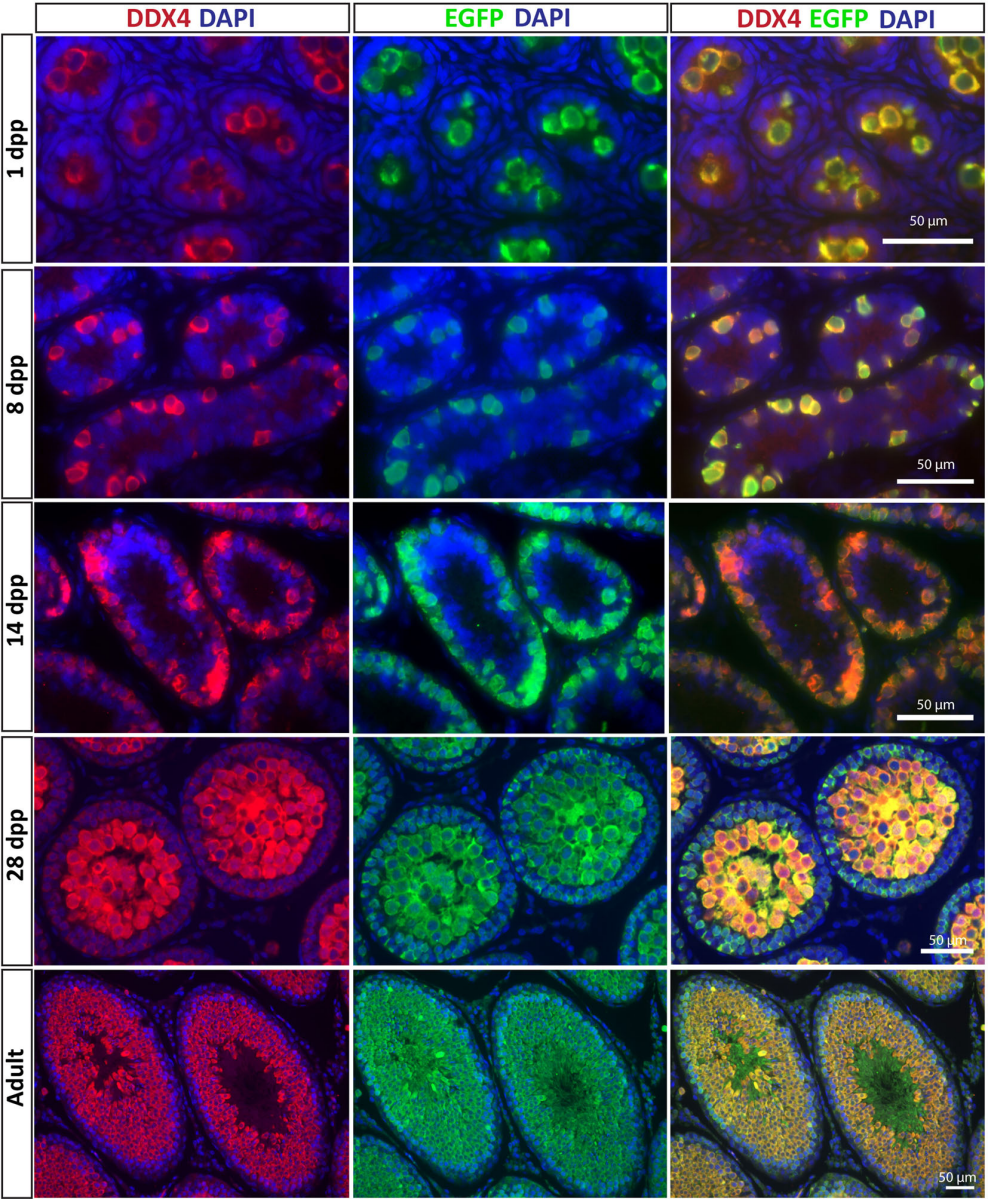
EGFP is expressed in germ cells throughout germ lineage development and spermatogenesis in postnatal transgenic DDX4-EGFP testes. Germ cells are identified by positive DDX4 staining at 1, 8, 14, and 28 dpp, and in adult testes (left column top to bottom). EGFP was consistently expressed in all DDX4-positive germ cells during postnatal development and during spermatogenesis (center column top to bottom), as confirmed in merged images (right column top to bottom). Scale bar = 50 μm.

In contrast, we observed EGFP expression in all DDX4-positive germ cells during postnatal male germline development, including gonocytes on postnatal day 1, premeiotic type A spermatogonia at postnatal day (PND) 8, and undifferentiated and differentiating germ cells during the first wave of spermatogenesis (PND 14 and 28) as well as in the adult testis (Figure 5). EGFP reporter expression only partially recapitulated DDX4 protein expression in the fetal testis. EGFP was not detectable in DDX4-positive germ cells of gestation day 15.5 testes or ovaries, but was observed in DDX4-positive germ cells of gestation day 18.5 (Supplementary Figure S2). Most DDX4-positive germ cells also expressed EGFP in the gestational day 18.5 testes, while only a portion (∼30%) of DDX4-positive germ cells also expressed EGFP in the gestational day 18.5 ovaries. No EGFP expression was observed in sections from wildtype testes or ovaries (Supplementary Figure S3). Testis tissue section from PND 8 and adult DDX4-EGFP rats were also coimmunostained for SALL4 and EGFP. At PND 8, all SALL4-positive cells were EGFP positive (Supplementary Figure S5A–C). In the adult testis (Supplementary Figure S5D–F), the majority of SALL4-positive cells were EGFP positive (arrow heads), but a few SALL4-positive cells were weak or negative for EGFP (arrows).

### Transplantable spermatogonial stem cells from DDX4-EGFP rats produce fluorescent green colonies of spermatogenesis in recipient testes

**Figure 6.**
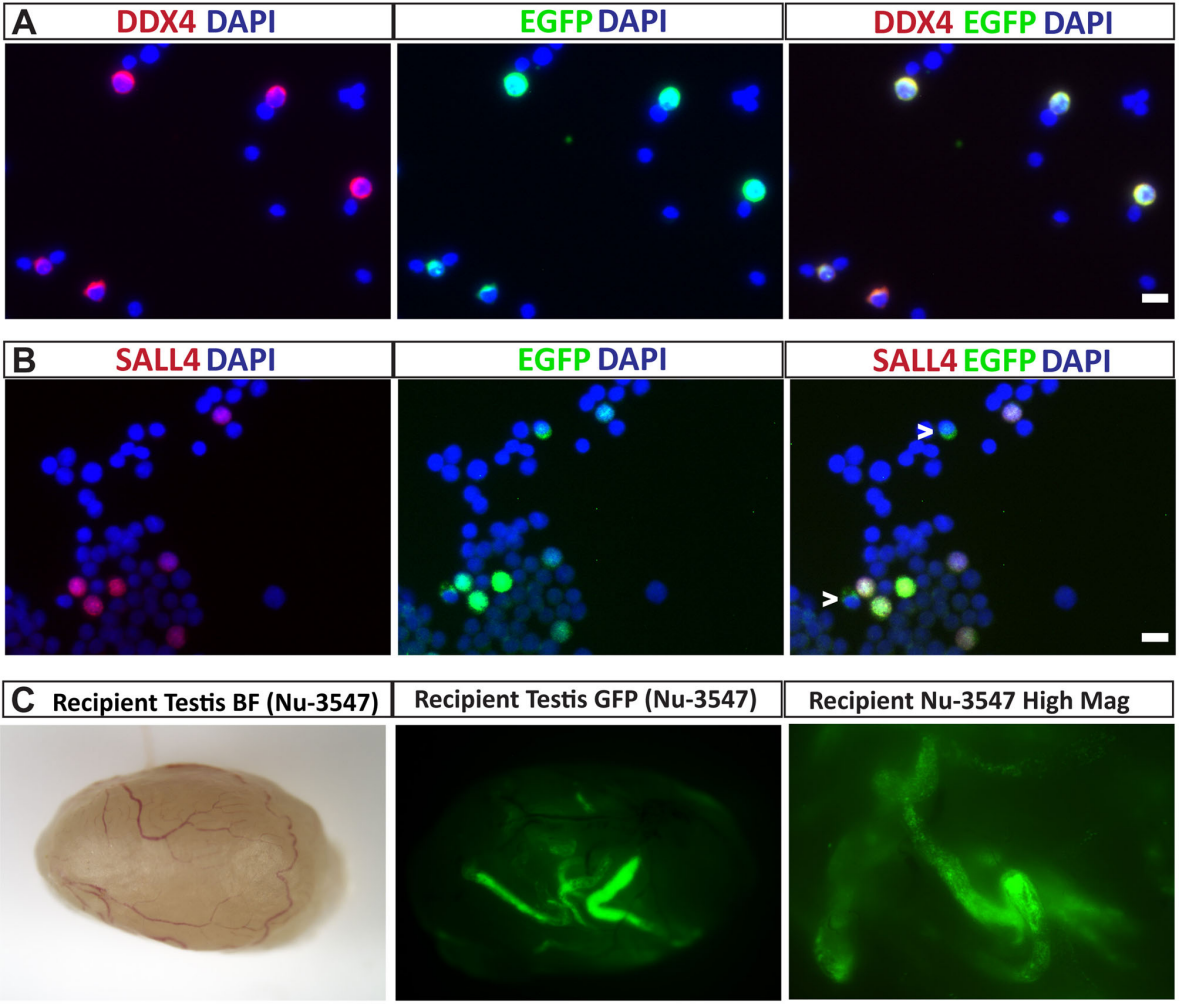
Transplanted SSCs from DDX4-EGFP rats produce green fluorescent colonies of spermatogenesis. (A) Single-cell suspensions from 14-day-old DDX4-EGFP testes were stained with anti-DDX4 antibody to identify germ cells, and anti-EGFP antibody to detect transgene expressing germ cells. All germ cells expressed EGFP. (B) The same single-cell suspension was costained with anti-SALL4 antibody to mark undifferentiated spermatogonia including SSCs. While most SALL4-positive spermatogonia at this age expressed EGFP, SALL4-negative/EGFP-positive germ cells were also observed (white arrowheads). Scale bars = 10 μm. (C) The same single-cell suspension as shown in A and B was transplanted into busulfan-treated infertile nude mouse recipient testes. Three months after transplantation, EGFP-positive colonies of donor-derived spermatogenesis could be observed, confirming the expression of the reporter gene in SSCs.

To determine whether the DDX4-EGFP reporter could be used to track the fate and spermatogenic potential of transplanted SSCs, we prepared single-cell suspensions from 14-day-old DDX4-EGFP rat testes and transplanted them into the testes of infertile nude mice (Figure 6). Prior to transplantation we used immunocytochemistry and coimmunofluorescence staining to characterize the donor cell suspension and confirm that EGFP is expressed by testicular germ cells, including stem and progenitor spermatogonia. Costaining with anti-EGFP and anti-DDX4 antibodies confirmed that most DDX4-positive germ cells also expressed EGFP (Figure 6A). SALL4 is a marker of stem and progenitor spermatogonia in monkey, human, and mice [67, 68], and is also conserved in rat spermatogonia (Supplementary Figure S4). Costaining of isolated cells with anti-EGFP and anti-SALL4 demonstrated that most EGFP-positive premeiotic germ cells from 14-day-old rats expressed SALL4. A few EGFP-positive cells that did not express SALL4 were also observed, which were presumably more differentiated spermatogonia (indicated by arrowheads in Figure 6B). Based on the immunocytochemistry results (Figure 5; Supplementary Figure S5), we hypothesized that functional stem cells could be recovered in the EGFP-positive fraction of DDX4-EGFP rat testes. When the EGFP-positive cells were injected into the seminiferous tubules of experimentally infertile, busulfan-treated nude mice, EGFP-positive colonies of donor-derived rat spermatogenesis were observed in all recipient testes 3 months after transplantation (Figure 6C).

### Isolation and long-term culture of DDX4-EGFP rat spermatogonial stem cells

**Figure 7.**
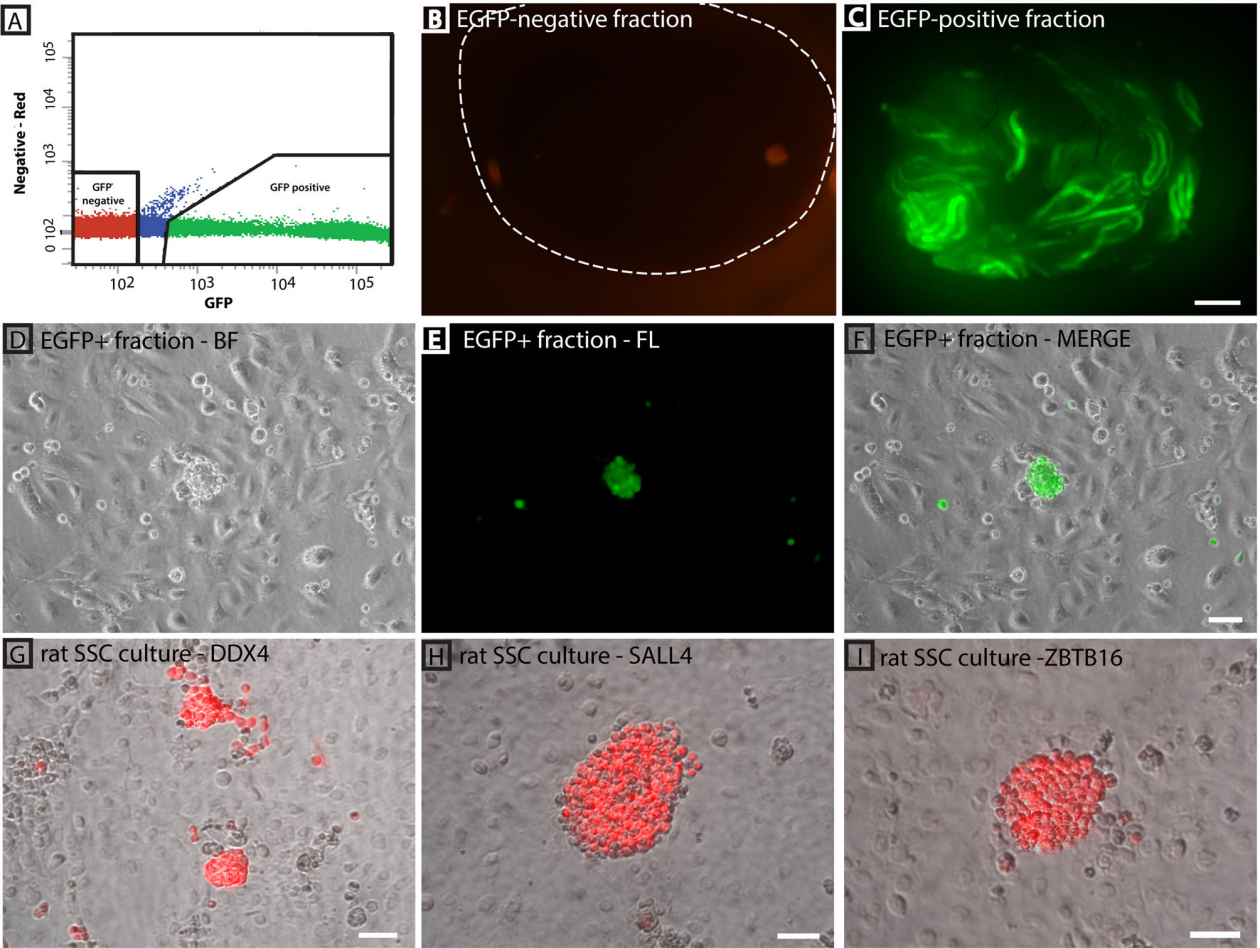
FACS isolation of DDX4-EGFP-positive spermatogonia for long-term rat SSC culture. (A) Sorting gates for separating EGFP-positive cells from EGFP-negative cells. Sorting of PND 10 testicular cells is shown as a representative example. (B) Transplantation of negative fractions into infertile recipient mouse testes did not result in colonies of spermatogenesis. (C) Abundant green fluorescent colonies of spermatogenesis were obtained when transplanting the same cell number of EGFP-positive sorted fractions into infertile recipient testes. Scale bar in B and C is 1 mm. (D–F) EGFP-positive germ cells isolated from PND 10 rat testes were cultured on STO feeder cells and maintained endogenous EGFP expression. Scale bar = 50 μm. (G–I) Long-term culture of rSSCs for 12 weeks was established that maintains DDX4 expression (germ cell marker), and SALL4 and ZBTB16 (two established spermatogonia marker). Scale bars = 50 μm.

EGFP-positive germ cells were isolated by FACS for transplantation into infertile recipient mice and to establish SSC cultures (Figure 7A). Direct transplantation of the EGFP-positive fraction into infertile recipient mouse testes resulted in the colonization of seminiferous tubules and the initiation of spermatogenesis, indicating the presence of SSCs in this cell population. In contrast, no green fluorescent colonies were found in recipients that received the EGFP-negative fraction (Figure 7B and C). In addition, green fluorescent spermatogenic colonies were more abundant compared to recipient testes that received the unsorted crude testicular cell suspension (see Figure 6C).

In addition to isolating functional stem cells, we hypothesized that sorting EGFP-positive germ cells from DDX4-EGFP rat testes would effectively eliminate testicular somatic cells from the cell suspension and facilitate establishment of rat SSC cultures. When the EGFP-positive fraction of cells from PND14 DDX4-EGFP rat testes were cultured on STO feeders in serum-free medium, DDX4-positive cells formed clusters of spermatogonia similar to those described previously for mouse and rat SSC cultures (Figure 7D–F, passage 2) and maintained endogenous EGFP expression over an extended period of time. Cultures could be propagated for up to 12 weeks (12 passages) and in addition to endogenous EGFP expression also maintained expression of DDX4, ZBTB16, and SALL4 in the colony-forming cells (Figure 7G–I, passage 12). We did not observe somatic cell overgrowth in those cultures. In contrast, cell cultures started from EGFP-negative fractions containing less than 1% DDX4-positive germ cells (based on DDX4 immunocytochemistry, n = 2) could not be maintained for more than 2 weeks due to an abundance of somatic cells (peritubular myoid fibroblasts, Sertoli cells) that continued to proliferate in vitro (data not shown).

## Discussion

We generated a transgenic DDX4-EGFP reporter rat that presents a powerful tool to study male germline development, SSCs, and spermatogenesis. A previous study reported the expression of ROSA-GFP exclusively in germ cells of the GCS-EGFP transgenic rat model after fortuitous integration of the transgene in a genetic region with several testis-specific transcribed loci [69]. EGFP expression in the GCS-EGFP rat model is due to the positional effect of the transgene. In contrast, our model is the first to express the EGFP reporter under the control of a GCS promoter region in rats. The EGFP expression pattern exhibited by postnatal DDX4-EGFP rats accurately reflects the reported expression pattern of endogenous DDX4, which has been widely used as a pan-germ cell marker in male mice and rats [59]. We observed that DDX4 is expressed in male germ cells at all stages of postnatal testis development, and in all spermatogenic cell types. In the adult testis, DDX4 expression is strongest in spermatocytes and spermatids, but it is also expressed at detectable levels in undifferentiated spermatogonia.

The DDX4-EGFP rat line can be used to address a variety of questions related to male germ cell biology. We show expression of EGFP in DDX4-positive gonocytes (or prospermatogonia) as early as gestation day 18.5 and at PND1, as well as type A premeiotic spermatogonia at PND 8. Thus, this rat can be used to study the earliest stages of spermatogenic lineage development, including the transition from gonocytes to type A spermatogonia and the establishment of the SSC pool. Isolating these specific cell types using FACS will allow researchers to dissect gene and protein expression in these distinct cell populations as they arise during development. This model has limited utility for studying fetal germ lineage development because EGFP reporter expression was not detectable in DDX4-positive germ cells until gestation day 18.5 in both males and females. This delayed expression of EGFP in fetal germ cells relative to endogenous DDX4 is likely a characteristic of the recombinant mouse DDX4 promoter used in this study because the same promoter exhibited a similar expression pattern in DDX4-Cre mice that were previously described [28]. Delayed EGFP expression relative to endogenous DDX4 expression could also be due to integration site effects or expression below the level of detection. However, since we used the same promoter described in the previous mouse study, we suspect that the expression pattern is characteristic of the recombinant promoter.

SSC culture has emerged as a powerful tool for dissecting the molecular mechanisms that regulate stem cell self-renewal and differentiation in mice. Methods to isolate spermatogonia and remove somatic cells were critical for the establishment of mouse SSC cultures, and GDNF and bFGF were required for long-term maintenance and expansion of SSCs, in vitro [70–72]. Mouse SSC cultures have provided insights about growth factors, signaling pathways, cell cycle regulators, transcriptional regulators, and small RNA molecules that coordinate SSC proliferation, survival, self-renewal, and differentiation [47, 73–76]. Spermatogonial stem cell transplantation provided the critical functional assay to assess the effects of in vitro manipulations on SSCs growth dynamics. Spermatogonial stem cell culture has now been extended to hamsters and rats where the requirement to remove testicular somatic cells and supplement with growth factors, including GDNF and bFGF, appears to be conserved [66, 77, 78]. The DDX4-EGFP reporter will facilitate isolation of spermatogonia and removal of somatic cells by FACS prior to the initiation of rat SSC cultures. Furthermore, our results indicate that the DDX4-EGFP reporter can be used to detect and quantify donor-derived spermatogenesis after transplantation into infertile recipients. Similarly, FACS has been used to isolate undifferentiated spermatogonia from GCS-EGFP rat testes [69], derive rat SSC cultures [77] and discover genes associated with stemness [79] and/or SSC survival [80].

EGFP did not faithfully recapitulate DDX4 expression in all follicles of the adult ovary. While EGFP was expressed by most DDX4-positive antral follicles, it was detectable in less than half of the DDX4-positive preantral (secondary/tertiary) follicles and only a few percent of DDX4-positive early (primordial/primary) follicles. These discrepancies could indicate that DDX4 protein level is simply below the level of detection in the early follicles and/or could indicate differences in translation or degradation of the DDX4 protein compared with the EGFP protein. Interestingly, we observed EGFP in a small percentage of preantral and antral follicles that did not express DDX4. The sex-dependent phenotype of DDX4 overexpressing mutant cell lines, and reports of heterogeneous *Ddx4* expression in the human ovary [81, 82], indicates that DDX4 function in the germline might be important for meiotic entry or progression, and thus, postnatal oocytes at different stages of development might show different levels of expression. This may contribute to the inconsistent results that we observed in ovaries from DDX4-EGFP females as well as male/female differences observed in this study. Perhaps distinct expression patterns of DDX4 and the EGFP proteins correlate with specific stages of meiotic progression. If this can be documented in future studies by costaining with stage-specific meiotic markers, it will expand the utility of the DDX4-EGFP rats for studying oocyte/follicle development.

The robust expression of EGFP in male germ cells, including stem and progenitor spermatogonia, makes this model a useful tool for future studies on rat SSCs and spermatogenic lineage development. This model combined with other existing/emerging rat resources such as the GCS-EGFP rat model, rat SSC cultures, rat embryonic stem cells, and CRISPR-Cas9 gene-editing tools provide unprecedented opportunities to study and manipulate the rat germline. EGFP expression in fetal germ cells (both sexes) and in postnatal oocytes/follicles of DDX4-EGFP rats did not faithfully recapitulate endogenous DDX4 expression. Additional studies are needed to dissect reason for those discrepancies and determine the potential utility of this model in those contexts.

## Supplementary data

Supplementary data are available at *BIOLRE* online.

**Supplementary Figure S1.** DDX4-EGFP genotyping. Tail DNA was isolated from pups born after embryo transfer and subjected to PCR to detect the EGFP transgene. Female #2738 was identified as transgenic and served as the founder animal for the established DDX4-EGFP line.

**Supplementary Figure S2.** EGFP expression in DDX4-positive germ cells in fetal testes and ovaries. EGFP expression was not detectable in DDX4-positive germ cells in gestation day 15.5 (A–F), but was observed in DDX4-positive germ cells in gestation day 18.5 (G–L).

**Supplementary Figure S3.** DDX4-EGFP costaining on wildtype rat testis and ovary sections. EGFP was absent in DDX4-positive adult male and female germ cells in wildtype testis and ovaries.

**Supplementary Figure S4.** SALL4 and ZBTB16 are conserved markers in rat spermatogonia. SALL4 (green) and ZBTB16 (red) were observed in rat spermatogonia along the basement membrane in the adult rat testis. Scale = 100 μm.

**Supplementary Figure S5.** SALL4 and EGFP expression in PND8 and adult rat testis. Postnatal day 8 and adult DDX4-EGFP rat testis tissue sections were costained for SALL4 and EGFP. (A–C) Postnatal day 8 testis staining for EGFP (green) and SALL4 (red). All SALL4-positive cells were also positive for EGFP. (D–F) Adult rat testis staining for EGFP (green) and SALL4 (red). The majority of SALL4-positive cells also expressed EGFP (arrowheads), but a few SALL4-positive cells exhibited weak or undetectable EGFP expression (arrows). Nuclei are stained blue with DAPI. Scale bar = 50 μm

**Supplementary Table S1.** Antibody table.

## Supplementary Material

Supplemental material Supplementary data are available at *BIOLRE* online.
**Supplementary Figure S1.** DDX4-EGFP genotyping. Tail DNA was isolated from pups born after embryo transfer and subjected to PCR to detect the EGFP transgene. Female #2738 was identified as transgenic and served as the founder animal for the established DDX4-EGFP line.
**Supplementary Figure S2.** EGFP expression in DDX4-positive germ cells in fetal testes and ovaries. EGFP expression was not detectable in DDX4-positive germ cells in gestation day 15.5 (A–F), but was observed in DDX4-positive germ cells in gestation day 18.5 (G–L).
**Supplementary Figure S3.** DDX4-EGFP costaining on wildtype rat testis and ovary sections. EGFP was absent in DDX4-positive adult male and female germ cells in wildtype testis and ovaries.
**Supplementary Figure S4.** SALL4 and ZBTB16 are conserved markers in rat spermatogonia. SALL4 (green) and ZBTB16 (red) were observed in rat spermatogonia along the basement membrane in the adult rat testis. Scale = 100 μm.
**Supplementary Figure S5.** SALL4 and EGFP expression in PND8 and adult rat testis. Postnatal day 8 and adult DDX4-EGFP rat testis tissue sections were costained for SALL4 and EGFP. (A–C) Postnatal day 8 testis staining for EGFP (green) and SALL4 (red). All SALL4-positive cells were also positive for EGFP. (D–F) Adult rat testis staining for EGFP (green) and SALL4 (red). The majority of SALL4-positive cells also expressed EGFP (arrowheads), but a few SALL4-positive cells exhibited weak or undetectable EGFP expression (arrows). Nuclei are stained blue with DAPI. Scale bar = 50 μm
**Supplementary Table S1.** Antibody table.
