# Supplementary material for: DDX4-EGFP transgenic rat model for the study of germline development and spermatogenesis
Source: Biol Reprod. 2017 Feb 20;96(3):707–19. doi: 10.1095/biolreprod.116.142828 (PMC5803776; doi:10.1095/biolreprod.116.142828)
Supplement: Supplemental material — Supplementary data are available at BIOLRE online. Supplementary Figure S1. DDX4-EGFP genotyping. Tail DNA was isolated from pups born after embryo transfer and subjected to PCR to detect the EGFP transgene. Female #2738 was identified as transgenic and served as the founder animal for the established DDX4-EGFP line. Supplementary Figure S2. EGFP expression in DDX4-positive germ cells in fetal testes and ovaries. EGFP expression was not detectable in DDX4-positive germ cells in gestation day 15.5 (A–F), but was observed in DDX4-positive germ cells in gestation day 18.5 (G–L). Supplementary Figure S3. DDX4-EGFP costaining on wildtype rat testis and ovary sections. EGFP was absent in DDX4-positive adult male and female germ cells in wildtype testis and ovaries. Supplementary Figure S4. SALL4 and ZBTB16 are conserved markers in rat spermatogonia. SALL4 (green) and ZBTB16 (red) were observed in rat spermatogonia along the basement membrane in the adult rat testis. Scale = 100 μm. Supplementary Figure S5. SALL4 and EGFP expression in PND8 and adult rat testis. Postnatal day 8 and adult DDX4-EGFP rat testis tissue sections were costained for SALL4 and EGFP. (A–C) Postnatal day 8 testis staining for EGFP (green) and SALL4 (red). All SALL4-positive cells were also positive for EGFP. (D–F) Adult rat testis staining for EGFP (green) and SALL4 (red). The majority of SALL4-positive cells also expressed EGFP (arrowheads), but a few SALL4-positive cells exhibited weak or undetectable EGFP expression (arrows). Nuclei are stained blue with DAPI. Scale bar = 50 μm Supplementary Table S1. Antibody table. [file bio142828_supp.pdf]

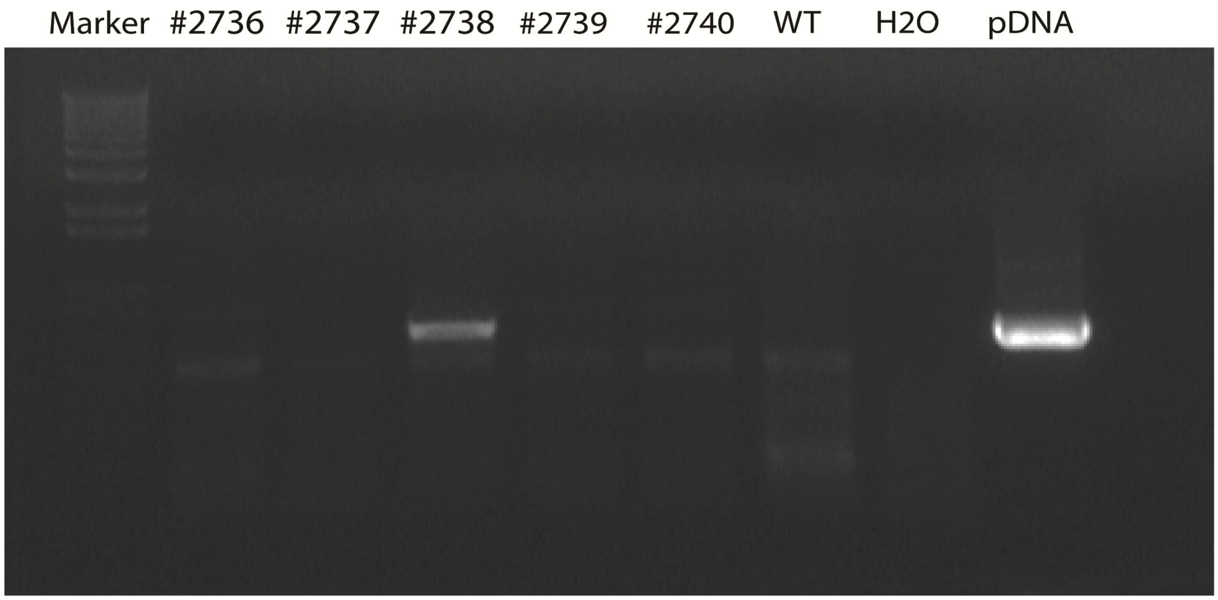

Figure S1: DDX4-EGFP genotyping. Tail DNA was isolated from pups born after embryo transfer and subjected to PCR to detect the EGFP transgene. Female #2738 was identified as transgenic and served as the founder animal for the established DDX4-EGFP line.

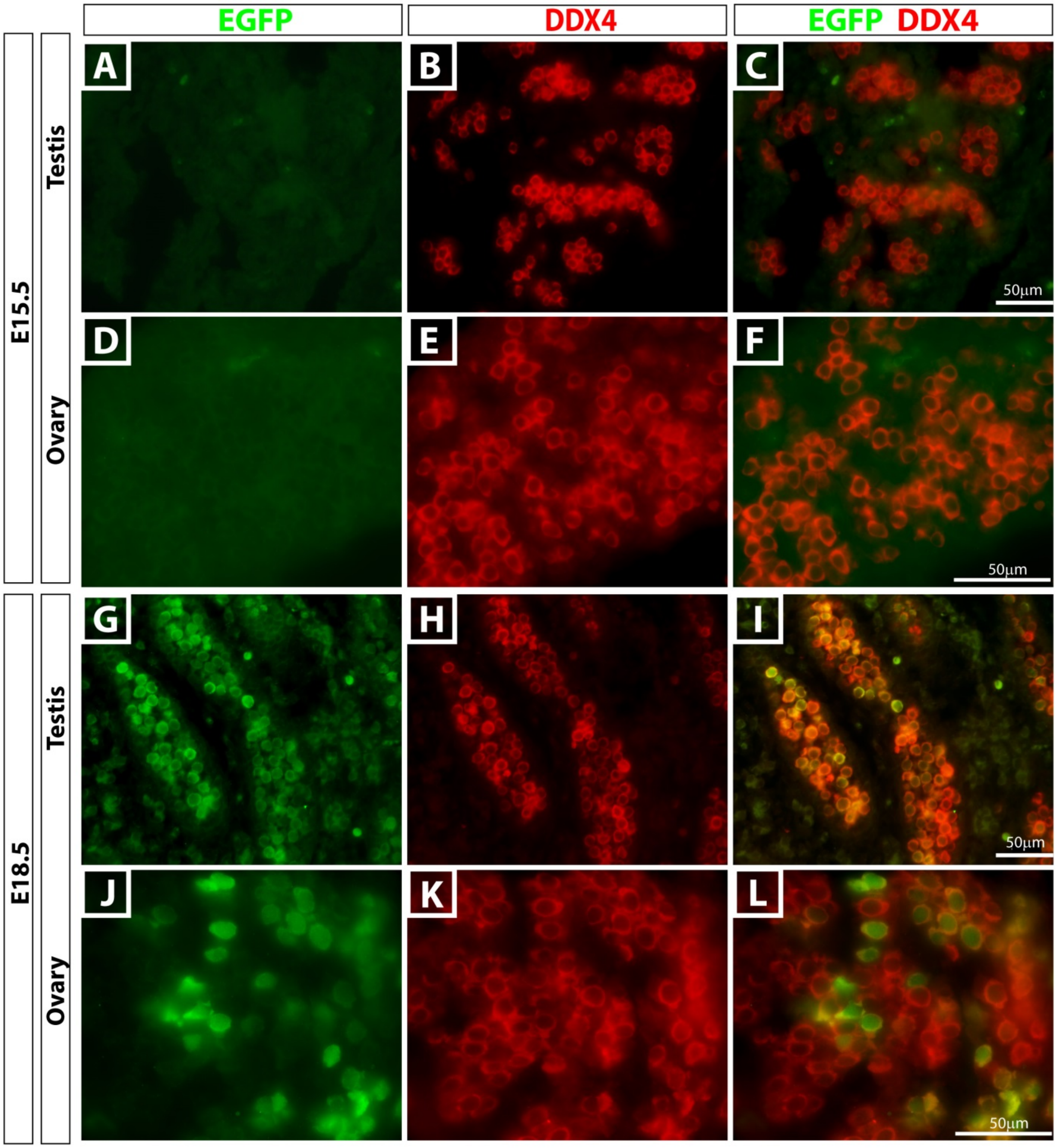

Figure S2: EGFP expression in DDX4 positive germ cells in fetal testes and ovaries. EGFP expression was not detectable in DDX4 positive germ cells in gestation day 15.5 (A-F), but was observed in DDX4 positive germ cells in gestation day 18.5 (G-L).

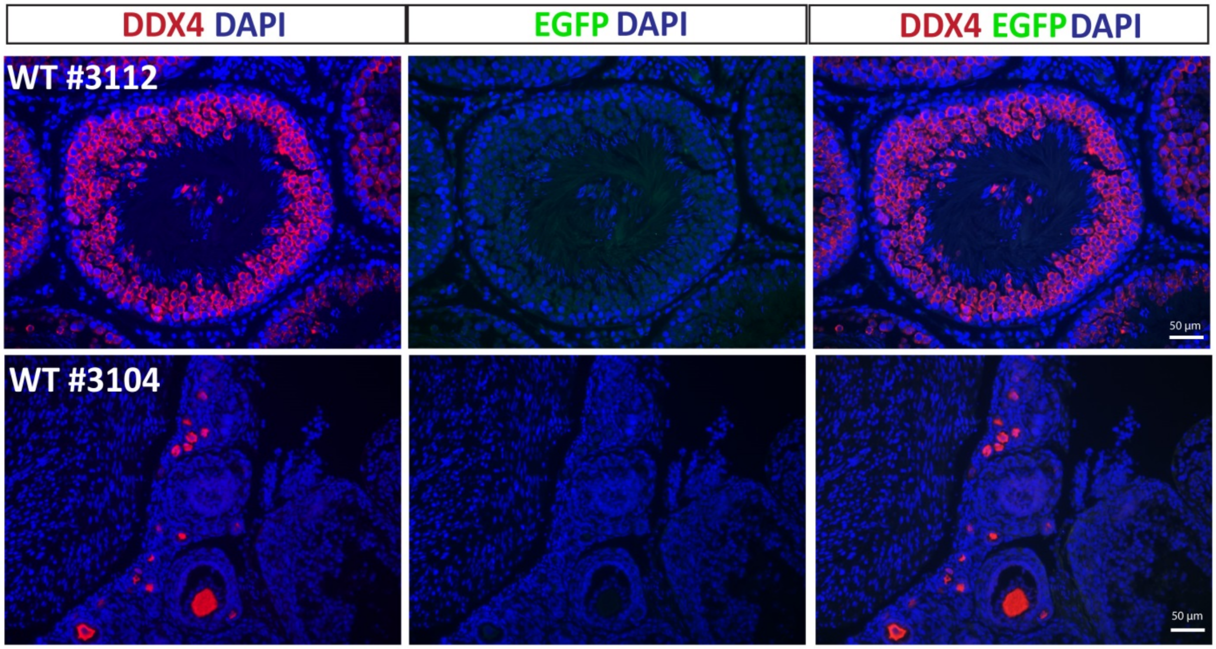

Figure S3: DDX4-EGFP co-staining on wildtype rat testis and ovary sections.

EGFP was absent in DDX4 positive adult male and female germ cells in wildtype testis and ovaries.

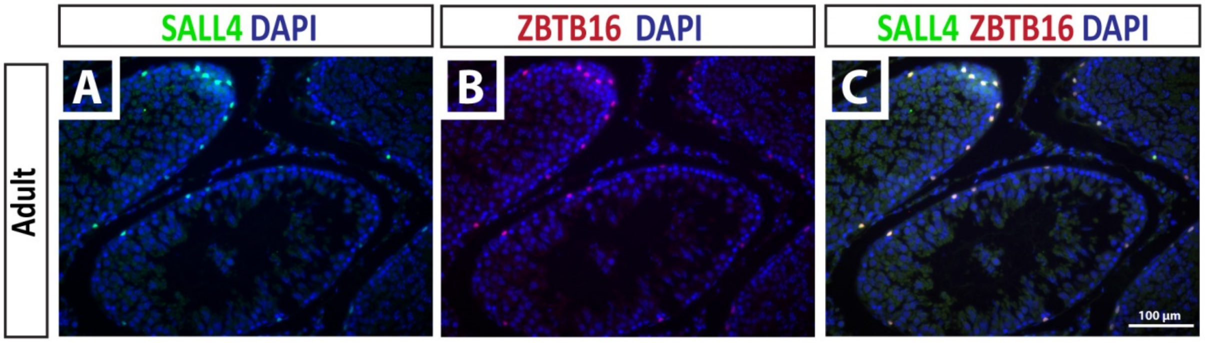

Figure S4: SALL4 and ZBTB16 are conserved markers in rat spermatogonia.

SALL4 (green) and ZBTB16 (red) were observed in rat spermatogonia along the basement membrane in the adult rat testis. Scale = 100 µm.

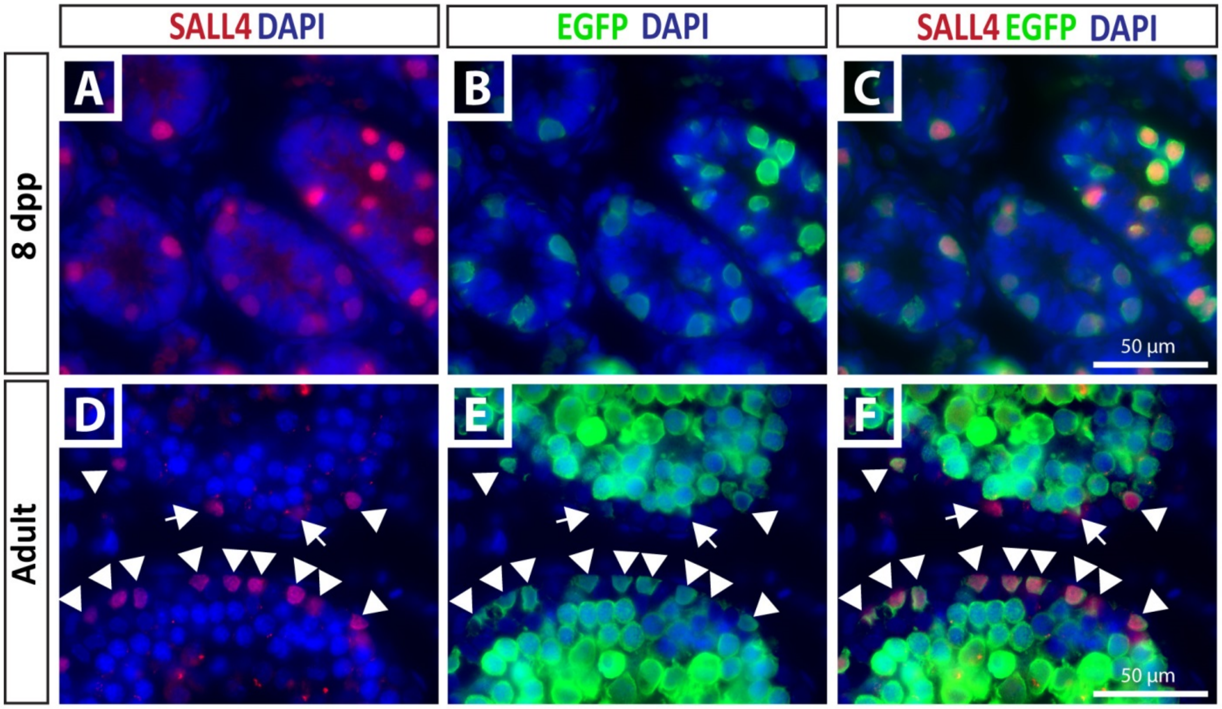

Figure S5: SALL4 and EGFP expression in PND8 and adult rat testis. Post-natal day 8 and adult DDX4-EGFP rat testis tissue sections were co-stained for SALL4 and EGFP. (A-C) Post-natal day 8 testis staining for EGFP (green) and SALL4 (red). All SALL4 positive cells were also positive for EGFP. (D-F) Adult rat testis staining for EGFP (green) and SALL4 (red). The majority of SALL4 positive cells also expressed EGFP (arrowheads), but a few SALL4 positive cells exhibited weak or undetectable EGFP expression (arrows). Nuclei are stained blue with DAPI. Scale bar = 50µm
